# Effect of preoperative CT angiography examination on the clinical outcome of patients with BMI ≥ 25.0 kg/m^2^ undergoing laparoscopic gastrectomy: study protocol for a multicentre randomized controlled trial

**DOI:** 10.1186/s13063-021-05887-1

**Published:** 2021-12-11

**Authors:** Cheng Meng, Shougen Cao, Xiaodong Liu, Leping Li, Qingsi He, Lijian Xia, Lixin Jiang, Xianqun Chu, Xinjian Wang, Hao Wang, Xizeng Hui, Zuocheng Sun, Shusheng Huang, Quanhong Duan, Daogui Yang, Huanhu Zhang, Yulong Tian, Zequn Li, Yanbing Zhou

**Affiliations:** 1 Department of Gastrointestinal Surgery, Affiliated Hospital of Qingdao University, No. 16 Jiangsu Road, Qingdao, China; 2 Department of Gastrointestinal Surgery, Shandong Provincial Hospital, Jinan, China; 3 Department of Gastrointestinal Surgery, Qilu Hospital of Shandong University, Jinan, China; 4 Department of Gastrointestinal Surgery, Qianfoshan Hospital of Shandong Province, Jinan, China; 5 Department of Gastrointestinal Surgery, Yantai Yuhuangding Hospital, Yantai, China; 6 Department of Gastrointestinal Surgery, Jining No.1 People’s Hospital, Jining, China; 7 Department of Gastrointestinal Surgery, Weihai Central Hospital, Weihai, China; 8 Department of Gastrointestinal Surgery, Dongying People’s Hospital, Dongying, China; 9 Department of Gastrointestinal Surgery, Rizhao People’s Hospital, Rizhao, China; 10 Department of Oncological Surgery, Weifang People’s Hospital, Weifang, China; 11 Department of Gastrointestinal Surgery, People’s Hospital of Jimo District, Qingdao, China; 12 Department of Gastrointestinal Surgery, Affiliated Hospital of Weifang Medical University, Weifang, China; 13 Department of Gastrointestinal Surgery, Liaocheng People’s Hospital, Liaocheng, China; 14 Department of Gastrointestinal Surgery, Weihai Municipal Hospital, Weihai, China

**Keywords:** Gastric cancer, CTA, Laparoscopic gastrectomy, Clinical outcomes, Study protocol, Randomized controlled trial

## Abstract

**Background:**

Gastric cancer, which is the fifth most common malignancy and the third most common cause of cancer-related death, is particularly predominant in East Asian countries, such as China, Japan and Korea. It is a serious global health issue that causes a heavy financial burden for the government and family. To our knowledge, there are few reports of multicentre randomized controlled trials on the utilization of CT angiography (CTA) for patients who are histologically diagnosed with gastric cancer before surgery. Therefore, we planned this RCT to verify whether the utilization of CTA can change the short- and long-term clinical outcomes.

**Method:**

The GISSG 20–01 study is a multicentre, prospective, open-label clinical study that emphasises the application of CTA for patients who will undergo laparoscopic gastrectomy to prove its clinical findings. A total of 382 patients who meet the inclusion criteria will be recruited for the study and randomly divided into two groups in a 1:1 ratio: the CTA group (*n* = 191) and the non-CTA group (*n* = 191). Both groups will undergo upper abdomen enhanced CT, and the CTA group will also receive CT angiography. The primary endpoint of this trial is the volume of blood loss. The second primary endpoints are the number of retrieved lymph nodes, postoperative recovery course, hospitalization costs, length of hospitalization days, postoperative complications, 3-year OS and 3-year DFS.

**Discussion:**

It is anticipated that the results of this trial will provide high-level evidence and have clinical value for the application of CTA in laparoscopic gastrectomy.

**Trial registration:**

ClinicalTrials.gov, NCT04636099. Registered November 19, 2020

**Supplementary Information:**

The online version contains supplementary material available at 10.1186/s13063-021-05887-1.

## Introduction

### Background and rationale

Since the first report of laparoscopic assisted-distal gastrectomy by Kitano in 1994 [1], it has gained great acceptance by surgeons and patients for treating surgically resectable gastric malignancy without bulky lymph nodes or lesions. It is a minimally invasive surgical procedure and has an enhanced recovery course and comparable oncological efficiency compared with that of open surgery, which has been demonstrated by some large-scale multicentre randomized controlled trials [2–7]. Although gastric cancer multimodal treatment has achieved excellent results, such as chemotherapy and immunity therapy, which have significant oncological advantages for treating advanced gastric malignant tumours, surgery is still the mainstream curative treatment for patients diagnosed with gastric cancer.

Radical lymphadenectomy associated with gastrectomy is an essential principle of the treatment of gastric cancer [8, 9]. The distribution of the lymph nodes along the vessels needs to be considered. The number of harvested positive lymph nodes is of great importance for predicting the long-term survival of patients, but even among skilled surgeons, lymphadenectomy is a challenging and tricky task, not only due to its technical difficulty in separating the lymph nodes from their surrounding tissues but also due to in some circumstances the difficulty of distinguishing bulky lymph nodes from the main perigastric arteries, such as the right gastric artery, common hepatic artery and spleen artery.

Because of the complexity and individual variations of the perigastric vessels, apart from the necessity of gaining familiarity with normal anatomical perigastric vessels before surgery, preoperative acquaintance with any aberrant anatomy of the vessels can help avoid intraoperative damage to the vessels, such as the left gastric artery and common hepatic artery, which can reduce intraoperative blood loss, protect the liver from dysfunction and achieve a fast postoperative recovery [10].

Although digital subtraction angiography (DSA) is regarded as the gold standard to detect the anatomical position and variations of the vessels [11], because of its invasive operation and relatively high expense, it has been replaced by CT angiography (CTA) in the field of gastrectomy for the purpose of leaning the variations of the perigastric vessels before surgery, and it can provide a three-dimensional image of the perigastric vessels [12, 13].

Some studies have already declared the usefulness of the application of CT angiography before surgery. Iino et al. demonstrated that preoperative acquisition of perigastric vessel information might contribute to the safe dissection of lymph nodes during laparoscopic gastrectomy [12]. According to the study of Mu et al. [14], the surgeon can have a good acquaintance of celiac artery on its origin, course and variation and vascular calcifications for the patients that underwent gastrectomy with the help of 64-MSCTA, and it is also recommended that CTA should be a preoperative conventional procedure in gastric cancer patients.

Based on a retrospective propensity score matching study [15], we proposed a novel classification of the perigastric vessels according to the processing of CTA images, which indicates that utilizing CTA can further improve short-term clinical recovery course including more harvest of lymph nodes and less estimated blood loss and operation time, compared with patients who received abdominal enhanced CT without CTA, especially for patients whose BMI ≥ 25 kg/m^2^. To the best of our knowledge, few multicentre randomized controlled prospective studies have focused on the application of CTA for patients who are diagnosed with gastric cancer before undergoing laparoscopic gastrectomy.

For further research, we planned this multicentre controlled trial, which is named after GISSG 20–01, to verify whether the use of CTA can improve the short- and long-term clinical outcomes of patients who undergo laparoscopic or robotic gastrectomy.

## Methods/design

### Objective

The aim of the GISSG20–01 study is to explore the short-term clinical recovery course and the long-term oncological effects of preoperative CTA for patients who undergo laparoscopic or robotic gastric cancer radical surgery.

### Trial design

The GISSG20–01 study is a multicentre, prospective, open-label clinical study in which patients who meet the inclusion criteria will be randomly assigned to an experimental group (CTA group) and a control group (non-CTA group) in a 1:1 ratio. In addition to the standard examinations in both groups, the patients in the CTA group will receive upper abdomen enhanced CT and CT angiography while the non-CTA group will receive upper abdomen enhanced CT only.

### Participant selection

**Fig. 1:**
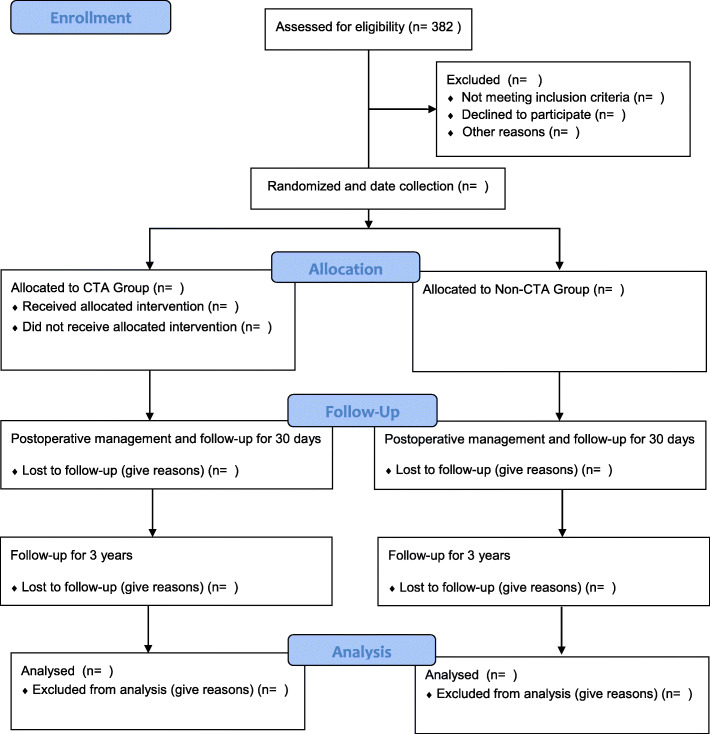
Study flowchart. CTA, CT angiography

Patients who are diagnosed with gastric cancer and plan to undergo laparoscopic or robotic gastrectomy will be enrolled from the 14 centres listed in Table 1. The launching conference was held online and was used to inform the participating staff about the details of the GISSG 20–01 trial. To obtain enough enrolled patients, all gastrointestinal surgeons in the participating centres were informed that the clinical trial was recruiting, and hospitalized patients were given the details about the trial for the purpose of enrolment. There are usually four ways to recruit patients: inpatients of gastrointestinal surgery, outpatients of gastrointestinal surgery, displaying poster at outpatient clinic or releasing recruitment information on the Internet. The enrolment of the first patient was started in November 2020, while it is anticipated that the deadline for the recruitment of the patients will be in November 2021. In the end, 382 patients who meet the inclusion criteria will be included in this trial. The study protocol and informed consent form were approved by the ethics review board of all research hospitals before the enrolment of any patients. Well-trained research doctors are responsible for introducing the main contents of the study to the patients and obtaining informed consent from patients who voluntarily participate in the trial in each centre. The flowchart that demonstrates the process of enrolment of the patients is shown in Fig. 1. The study protocol was revised to version 1.3 in December 2020. Any major modifications to the study protocol that may have a significant influence on the execution of the trial, including changes in the inclusion or exclusion criteria, number of samples or the study design, will require a formal amendment to the protocol. Such major modifications will be agreed up on by the GISSG (Gastrointestinal Surgery Study Group) and approved by the ethics review board. Protocol modifications are transmitted through the research meeting, and the electronic manual is distributed to the sub-centre researchers. An integral checklist of items in accordance with SPIRIT (Standardized Protocol Items: Recommendations for Intervention Trials) (2013 version) was supplied in additional file 1.

**Table 1:** The 14 participating centres

Number	Centre	Department	Investigator
01	The Affiliated Hospital of Qingdao University	Gastrointestinal Surgery	Yanbing Zhou
02	Shandong Provincial Hospital	Gastrointestinal Surgery	Leping Li
03	Qilu Hospital of Shandong University	Gastrointestinal Surgery	Qisi He
04	Affiliated Hospital of Shandong First Medical University	Gastrointestinal Surgery	Lijian Xia
05	Yantai Yuhuangding Hospital	Gastrointestinal SIurgery	Lixin Jiang
06	Shandong Jining No.1 People's Hospital	Gastrointestinal Surgery	Xianqun Chu
07	Affiliated Hospital of Weifang Medical University	Gastrointestinal Surgery	Quanhong Duan
08	Weifang People's Hospital	Gastrointestinal Surgery	Zuocheng Sun
09	Dongying People's Hospital	General Surgery	Hao Wang
10	Weihai Municipal Hospital	Gastrointestinal Surgery	Huanhu Zhang
11	Weihai Central Hospital	Gastrointestinal Surgery	Xinjian Wang
12	Rizhao People's Hospital	General Surgery	Xizeng Hui
13	People's Hospital of Jimo District, Qingdao	Gastrointestinal Surgery	Shusheng Huang
14	Liaocheng People's Hospital	Gastrointestinal Surgery	Daogui Yang

### Inclusion and exclusion criteria

The inclusion criteria are as follows: (1) patients with a pathological diagnosis of gastric adenocarcinoma by gastroscopy, (2) patients aged between 18 and 75 years old, (3) patients with a body mass index (BMI) not less than 25.0 kg/m^2^, (4) tumour clinical stage evaluated by CT imaging (T1~T4a, N0~3, M0), (5) patients with an Eastern Cooperative Oncology Group (ECOG) score 0–1 points, (6) the surgical approach is laparoscopic or robotic surgery, and (7) patients who are willing to participate in the study and sign the informed consent form.

The exclusion criteria are as follows: (1) patients whose tumour clinical stage conformed to T4b or M1 and whose tumour was found to be unresectable during the operation; (2) patients who suffered from a history of other malignant tumours or tumours of low malignant potential (such as giant cell tumours of the bone, pseudomyxadenoma of the appendix, invasive fibroma); (3) patients who have other serious systemic diseases and could not tolerate the trauma of surgery; (4) patients with non-adenocarcinoma-type malignant tumours verified by pathology after surgery; (5) patients with residual gastric cancer; (6) patients who are allergic to iodine contrast agents; (7) patients who received neoadjuvant therapy before surgery; (8) pregnant or lactating patients; and (9) patients who are participating in other clinical trials.

### Randomization

In this trial, eligible patients will be randomly assigned to either the CTA group or the non-CTA group in a 1:1 ratio. A central dynamic, stratified strategy was adopted for the aim of randomization. The sequence of randomization was generated by a certain doctor who was independent of this trial using the method of Pocock-Simon minimization by SAS 9.3 (SAS Institute Inc., Cary, NC, USA) and stratified by participating site (14 hospitals) and surgical approach (laparoscopic or robotic). The above information provided by the participating centres was submitted to the data centre at the Department of Gastrointestinal Surgery, Affiliated Hospital of Qingdao University, China, where central randomization was executed. Consequently, the allocation information will then be sent to each participating site. The allocation procedure was not blinded to the investigators or patients but was masked for data collection and analysis.

### Sample size computation

A non-inferiority hypothesis test was adopted in this study. Based on a previous retrospective study that showed that the mean ± standard deviation of the estimated blood loss during the operation in the CTA group and non-CTA group was 72.5 ± 66.4 ml and 93.5 ± 88.4 ml [15], respectively, we calculate that 382 patients were required to participate in this study (191 patients in the experimental group and 191patients in the control group) providing a significant level of *α* = 0.05 using a one-sided two-sample *t* test, a power of 1-β = 80%, and a maximum dropout rate of approximately 10%. The sample size computation was performed using PASS 11 (NCSS, LLC. Kaysville, UT, USA).

### Outcomes

The primary endpoint of this trial is the volume of blood loss that occurs during the surgery. The second primary endpoints are as follows: (1) the harvested number of lymph nodes is defined as the dissection number of lymph nodes through the standard extent of lymph node dissection, which is conducted with the principal of lymph node dissection guided by the 2018 version of Japanese Gastric Cancer Treatment Guidelines; (2) postoperative recovery course, which refers to the items of time to first ambulation, flatus, liquid diet and so on; (3) hospitalization costs are defined as the total expenses during hospitalization brought on by treatment and care; (4) length of hospitalization days, which is defined as from the day of surgery to the day of discharge; and (5) the severity of postoperative complications, which include anastomotic leakage, pancreatic fistula, and pulmonary or abdominal infection, and are evaluated by the classification of Clavien–Dindo [16]. Complications were recorded when they were worse than grade II. Moreover, 3-year OS (overall survival) and 3-year DFS (disease-free survival) will also be followed up and recorded.

### Interventions and CTA protocol

Both laparoscopic and robotic gastrectomy procedures associated with the standard extent of lymph node dissection were performed by reference to the 2018 version of Japanese Gastric Cancer Treatment Guidelines [9], and the resection range of the stomach, which is based on the Japanese Gastric Cancer Association classification [17], is according to lesion location and size, which is described in gastroscopy reports. Although there are three classic types of digestive tract reconstruction named after Billroth I, Billroth II and Roux-en-Y, the last type was recommended in this study. The TNM staging system was adopted from the eighth version of the American Joint Committee on Cancer (AJCC). Perioperative management will follow the basic principles of enhanced recovery after surgery in both groups. Chemotherapy guidance and follow-up will be conducted for patients with non-early gastric cancer based on 2021 version of NCCN gastric cancer clinical practice guidelines in oncology [18]. This study does not make uniform mandatory requirements for chemotherapy regimens and cycles, but postoperative adjuvant chemotherapy needs to be recorded in the CRF table. This study does not recommend routine postoperative adjuvant radiotherapy. Analgesic and antiemetic drugs are allowed in treatment measures, and the side effects of drug treatment should be recorded in the CRF.

A preoperative mandatory abdominal enhanced CT scan will be performed in both the experimental group and the control group to evaluate the clinical stage of the tumour. Moreover, compared with the non-CTA group, the patients in the CTA group will be asked to undergo the CTA examination once they are randomly assigned to the experimental group. To improve adherence to the intervention protocols, the patients will be fully informed of the potential short-term clinical recovery course and long-term oncological effects. Regular research meetings are held to provide relevant study guidance for researchers. For the researchers, there are mainly three ways to enhance adherence to intervention protocols including researcher training, regular researcher meetings and regular visits to the sub-centres. Abdominal enhanced CT will be performed using a 64-detector row CT scanner in this study, while the type of CT equipment is according to what is available in each participating centre. The procedure of the abdominal enhanced CT was set to ensure the same standard for the reconstruction of the perigastric arteries across centres. Fasting no less than 8 h before the examination is required, and an injection of 10 mg anisodamine 10 min before the examination is necessary for the purpose of reducing gastric distention and motility. Nonionic iodinated contrast material will be used as the contrast agent, injected at a speed of 4.0 ml/s by an automatic injector, and its volume is based on the weight of the patient; for example, for patients weighing 60 kg or less, the lower limit is 120 ml, and for patients weighing more than 75 kg, the upper limit is 150 ml.

### For qualification of the surgeons

To ensure the quality of the clinical trial and guarantee the safety of patients, some essential principles should be achieved before a surgeon participates in the study. Apart from the surgeon performing at least 100 laparoscopic or robotic gastrectomies, which means that the learning curve has been completed, two surgical videos are needed to be submitted to a surgical treatment quality control committee that consisted of two senior surgeons independent of the trial to verify that the surgeon meets the research requirements.

### For the quality of the CTA image

The scanning range of the abdominal enhanced CT will be the upper abdomen, from the top of the diaphragm to the level of the inferior mesenteric artery, and the region of interest (ROI) is at the level of the celiac artery. Since the quality of CTA is of utmost importance in this trial, we set up criteria to ensure the image quality: (1) there was no bundle sclerosis artefact produced by metal foreign bodies in vitro, which obviously affected the display effect of the arteries; (2) there was no motion artefact caused by motion displacement; and (3) MPR, VR, MIP and other three-dimensional reconstruction techniques could display the perigastric artery clearly. After the reconstruction of the CTA, the anatomy of the perigastric arteries will be captured to evaluate their origins and branches, such as the celiac artery and the hepatic artery, by two senior radiological specialists. In addition, to decrease potential damage to the perigastric arteries, the surgeon and the surgical team should have a good acquaintance with the anatomy of the perigastric arteries and the perigastric artery types, which were divided into seven types based on our previous proposal for a novel classification of the perigastric arteries, which is recorded in the CRF.

### Adverse events

Adverse events are defined as unfavourable and negative clinical outcomes occurring when patients receive medical care. Any adverse events associated with the CTA and the surgery will be recorded and treated properly. Nonionic iodinated contrast material will be used in this trial, and serious allergic reactions, including hypotension, dyspnoea and anaphylactic shock, have not been observed for this contrast agent. If an allergic reaction does occur, withdrawal of the enhanced CT examination from the patient will be documented as an adverse event. Meanwhile, patients can withdraw from the clinical trial without any responsibility. All participants were guaranteed by the national medical security system in the event of suffering any unpredictable injury during the trial. To monitor the enrolled data and ensure the safety, effectiveness and integrity of this clinical trial, an independent data and safety monitoring committee (DSMC) that consists of two senior surgeons, one statistician and one medical ethics expert was set up. The DSMC will evaluate the progression of patient enrolment and make suggestions to the sponsor regarding whether to continue the trial as planned, continue after modifying the protocol or suspend or terminate the trial.

### Postoperative care

The patients will be assessed by the doctor in charge twice a day, and any discomfort or surgical complications should be appropriately explained and handled; moreover, this management will be recorded in the CRF. The patients should be discharged from the hospital when they meet these criteria: postoperative pain is well controlled with or without oral analgesics, the body temperature is no more than 37 °C, the postoperative complications are well managed, oral semiliquid food is tolerated and the patient can sustain daily walking activity.

### Follow-up

**Fig. 2:**
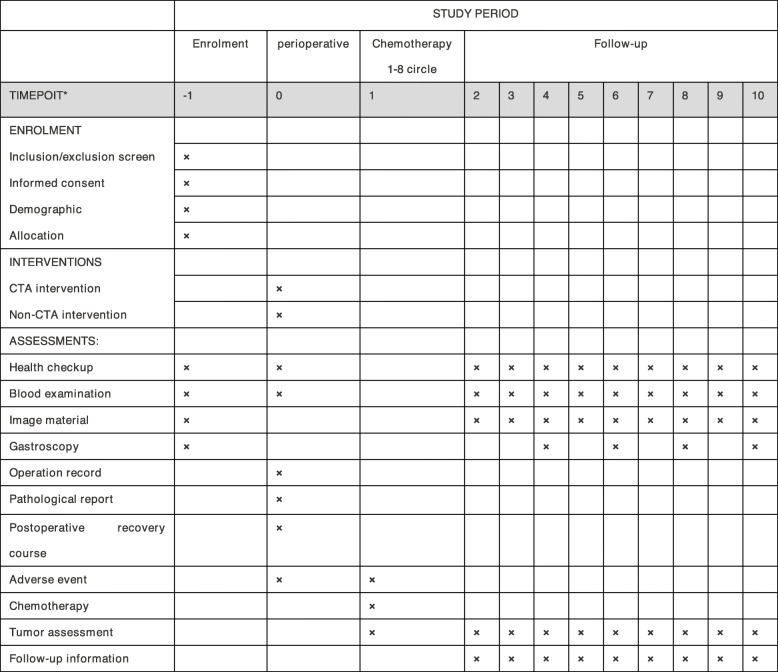
The items of enrolment, interventions and assessments in the flowchart. The symbol of × represent that the program needs to be collected. −1, 2 weeks before operation; 0, perioperation; 1, postoperative adjuvant chemotherapy time; follow-up 2~10 refers to time points that listed in the following: 2, 1 months after surgery; 3, 3 months after surgery; 4, 6 months after surgery; 5, 9 months after surgery; 6, 12 months after surgery; 7, 18 months after surgery; 8, 24 months after surgery; 9, 30 months after surgery; 10, 36 months after surgery

A specified follow-up team will be arranged for the patient after surgery at each participating centre, and the main follow-up methods include outpatient visits, telephone visits or mail visits. The postoperative recovery course, which includes a short-term recovery course and the long-term oncological effects and adjuvant therapy, will be assessed, as shown in Fig. 2. For the first year after surgery, the patient needs to be reviewed every three months. For the next 2–3 years, the patient needs to be reviewed every 6 months. The follow-up programme includes a physical examination, laboratory tests that consist of routine blood tests, liver and kidney function tests, electrolytes, and digestive tract tumour markers (including the examination of CEA, CA125, CA199 and CA724). Moreover, gastroscopy and chest and abdominal CT will be performed once a year and every 6 months, respectively. MRI or PET-CT will be used when a tumour recurrence or metastasis is suspected. In addition, the survival status of the patients will be noted in the CRF, and patients who undergo a follow-up of 36 months are essential for the study.

### Data collection and analysis

An a priori designed and coincident case report form (CRF) will be used to record the information required by the study, such as baseline characteristics, laboratory data, perioperative clinical recovery course and long-term oncological outcomes, and a designated clinical investigator is responsible for the collection of data at each centre. Moreover, to keep track of changes to the data, any correction to the raw data requires signing the date and the investigator’s name. A monthly data check that includes data summarized by each centre, abnormal data and delayed data in progress tracking is required during the trial period.

Continuous variables will be presented as the mean ± standard deviation, while classification variables will be described as numbers (*N*) and percentages (%) to express the difference between two groups. To compare continuous variables for the two groups, Student’s *t* tests or Mann–Whitney *U* tests will be performed, and categorical variables will use the *χ*^2^ test or Fisher’s exact test. OS and DFS, defined as the time from surgery to death and the time from surgery to tumour recurrence or death from any cause, respectively, will be assessed by the Kaplan–Meier method with the log-rank test to analyse the differences in the survival curves. A Cox proportional hazards regression model with a 95% confidence interval will be used to perform univariate and multivariate analyses. Subgroup analyses will also be performed, with participants stratified by BMI (25.0 to 29.9 kg/m^2^, ≥ 30 kg/m^2^), to verify the effect of CTA on short-term and long-term clinical outcomes in overweight and obesity patients between CTA group and non-CTA group. For missing data, multiple imputation was used to obtain complete datasets. A *P* value less than 0.05 will be considered statistically significant. The 25th version of SPSS (SPSS Inc., Chicago, IL, USA) will be used to analyse the data.

## Strengths and limitations

The strength of this study is that it is the first multicentre randomized controlled trial to evaluate the feasibility and benefits of the application of preoperative CTA for the short-term clinical recovery course and long-term oncological effects in patients who will undergo laparoscopic-assisted radical resection for gastric cancer. A limitation is that the subjective consciousness of the surgeon may lead to deviations in the results because this is an open-label trial.

## Discussion

Minimally invasive surgery, such as laparoscopic and robotic techniques, has gained great popularity due to its minimal trauma in the treatment of gastric cancer in recent decades [3, 19–21]. However, laparoscopic surgery lacks three-dimensional anatomical vision and distinguishes the relationship between intra-abdominal organs and vessels in spatial conformation compared with open surgery. Meanwhile, the local magnifying effect of laparoscopy makes it easy for the surgeon to lose their overall judgement of the adjacent relationship of perigastric tissues, and it is difficult to dissect the celiac artery and its branches accurately.

CT angiography (CTA), which is noninvasive and easy to perform, is a combination of CT enhancement technology and thin-layer, large-scale, fast scanning technology [22]. It can clearly show the details of blood vessels in all parts of the body through reasonable postprocessing of reconstruction. It is of great value for vascular variations and vascular diseases and displays the relationship between lesions and vessels [14, 23]. Through this technology, we can clearly understand the anatomical conditions of gastric blood vessels and related blood vessels and provide better and more detailed lesion information for planning gastric cancer surgery.

The possibility of unanticipated bleeding may be increased in the process of lymph node dissection among obese patients underwent laparoscopic or robotic gastrectomy. However, under the application of CTA, unpredictable perigastric artery damage and intraoperative bleeding, especially for the celiac trunk and its three major branches (left gastric artery, splenic artery and common hepatic artery), could be reduced during lymph node dissection with perigastric arteries navigation. Natsume et al. demonstrated that the visualization of the precise anatomy around the gastric with the application of dual-phase 3D CT is useful and essential modality, which may contribute to assist surgeons to reduce the intraoperative blood loss during laparoscopic gastrectomy [24]. Shen et al., who proposed a novel perigastric arteries classification, showed that compared with patients with non-high risk type vascular variation, patients with high risk type vascular variation have a high incidence of potential intraoperative vascular injury during laparoscopic radical gastrectomy [15].

Some related literatures have confirmed that the utility of CTA before laparoscopic gastrectomy can improve short-term clinical recovery course and play a vital role in guiding laparoscopic gastric cancer surgery [13, 15, 25]. Although the application of CTA was examined in our study only for the determination of perigastric artery anatomy or its variation in patient being received laparoscopic gastrectomy, depiction of visceral anatomy is also beneficial for the patient who undergoes pancreatic, hepatobiliary and liver transplantation surgery. Corinne et al. revealed that the CTA can be used to classify both normal anatomy and rare variation of celiac and haptic artery, which is essential for the surgical management for the patients with pancreatic and hepatobiliary malignancies [26]. Saylisoy et al. reported that multislice CT angiography can provide the detailed information of hepatic artery structure of candidates prior to the liver transplantation, leading into the possibility of decreasing the difficulty of surgery, reducing the incidence of contraindications and enhancing the chance of successful technical performance [27].

Overall, the application of CTA before surgery can provide information on the variation of perigastric vessels, contribute to the development of a surgical formula in advance and guide lymph node dissection. It is expected that the results of this trial can provide evidence and have clinical value for the application of CTA in laparoscopic gastrectomy, which is consistent with precise surgery.

## Trial status

Patient recruitment is still ongoing and the trial is still in the stage of collecting data at each participating site. To better execute this trial and ensure the safety of the patients, the study protocol was modified to version 1.3.

## Supplementary Information

**Additional file 1.** Standard Protocol Items: Recommendations for Interventional Trials (SPIRIT) 2013 checklist.

## Data Availability

The final datasets that are collected and analysed by this clinical trial will be obtained from DSMC after the publication of this trial.

## Abbreviations

BMI: Body mass index
CT: Computed tomography
CTA: CT angiography
MRI: Magnetic resonance imaging
PET-CT: Positron emission tomography CT
CRF: Case report form
OS: Overall survival
DFS: Disease-free survival
